# IRF7 inhibits the Warburg effect via transcriptional suppression of PKM2 in osteosarcoma

**DOI:** 10.7150/ijbs.65255

**Published:** 2022-01-01

**Authors:** Zhikun Li, Mei Geng, Xiaojian Ye, Yunhan Ji, Yifan Li, Xiangyang Zhang, Wei Xu

**Affiliations:** 1 Department of Orthopedics, Tongren Hospital, School of Medicine, Shanghai Jiao Tong University, Shanghai 200336, China.; 2 Department of Oncology, Rui Jin Hospital Affiliated to Shanghai Jiao Tong University School of Medicine, Shanghai 200025, China.

**Keywords:** IRF7, Aerobic glycolysis, Warburg effect, Osteosarcoma, PKM2

## Abstract

Osteosarcoma (OS) is a malignant bone tumor among adolescents and young adults. IRF7 belongs to the transcription factor family of interferon regulatory factors (IRFs) and has previously been described to function as a tumor suppressor in multiple cancer types. However, the biological functions and cellular mechanism of IRF7 in OS remain elusive. In this study, by quantitative real-time PCR (qRT-PCR) and western blotting, we found that IRF7 was downregulated in OS, and the higher expression of IRF7 was correlated with a better survival prognosis. Moreover, loss-of-function and gain-of-function studies have proved the critical functions of IRF7 in suppressing aerobic glycolysis of osteosarcoma cells as evidenced by glucose uptake, lactate production, extracellular acidification rate, and oxygen consumption rate. Mechanistically, IRF7 inhibited the expression of key glycolytic gene PKM2 via direct transcriptional regulation. Moreover, the *in vitro* and *in vivo* tumor-suppressive roles of IRF7 were uncovered in OS and these effects were largely glycolysis-dependent. Therefore, our study unveils a previous unprecedented role of IRF7 in glucose metabolism reprogram and suggests that IRF7 might serve as a potential therapeutic target for patients with OS.

## Introduction

Osteosarcoma (OS) is the most prevalent primary musculoskeletal malignancy and is most observed in the medullary ends of the long bones. OS predominantly occurs in children and teenagers with 10 to 25 years as the major onset age. OS progresses rapidly and has high morbidity and mortality 1. Despite remarkable progress in clinical treatment including surgical resection, neoadjuvant chemotherapy, and immunotherapy, the prognosis in OS patients remains poor due to distant metastasis 2. Abnormal gene expression contributes to diverse malignant phenotypes of OS cells and is critical factor affecting prognosis. Deciphering the molecular mechanism underlying OS development and progression may provide novel strategies for the treatment efficacy of OS and therapy to improve patients' prognosis 3.

Cancer cells preferably metabolize more glucose via aerobic glycolysis to generate the energy instead of mitochondrial oxidative phosphorylation even in the presence of sufficient oxygen, a phenomenon known as the Warburg effect. Warburg effect has emerged as a key hallmark of cancers including pancreatic cancer, liver cancer, breast cancer, and OS 4-7. Besides providing energy for cellular processes, the Warburg effect is essential for biosynthesis building blocks to sustain rapid cancer cell proliferation 8. Therefore, the elevated Warburg effect is associated with larger tumor size and predicts an unsatisfactory clinical outcome in cancer patients. Previously, many reports have well documented several transcription factors in regulating the Warburg effect, such as TP53 9, HIF1α 10, c-Myc 11, FOXK1/2 12, and SIX1 13. However, the transcriptional regulation of the Warburg effect in OS remains largely unknown.

Interferon regulatory factors (IRFs) belong to the transcription factor family and play important roles in immune regulation, cell cycle regulation, cell differentiation, and cell apoptosis 14, 15. IRFs were originally recognized for their notable roles in innate immune responses. Interestingly, emerging studies reveal diverse roles of IRF family members in tumor biology, including but not limited to tumor growth, cell invasion, angiogenesis, and chemotherapy resistance 16-18. Overall, IRFs are associated with tumor-suppressive properties in human cancer 19. For instance, loss of a negative feedback loop between IRF8 and AR in prostate cancer facilitates tumor growth and enzalutamide resistance 20; silencing of IRF4 in B cells accelerates the development of chronic lymphocytic leukemia 21; IRF5 inhibits replication of hepatitis C virus (HCV) and suppresses HCV-associated hepatocellular carcinoma 22. However, the potential roles and cellular mechanisms of IRF family members in OS remain elusive.

In this study, we investigated the expression pattern and prognostic value of IRF family members in OS and found that IRF7 was downregulated in OS and predicted a better prognosis. Upon genetic manipulation of IRF7 expression, we discovered that IRF7 inhibited aerobic glycolysis and repressed OS tumor growth. Furthermore, we demonstrated that IRF7 acted as a transcription factor to repress the translation of the key glycolytic factor PKM2.

## Materials and methods

### Cell culture and reagents

Human osteosarcoma cell lines, including KHOS, MNNG-HOS, MG63, Saos2, U2OS, a normal human osteoblast line hFOB1.19 were purchased from American Type Culture Collection (ATCC, Manassas, VA, USA) or Cell Resource Center of Shanghai Institutes for Biological Sciences (Shanghai, China). All cell lines were cultured in IMDM or DMEM containing 10% (v/v) fetal bovine serum (FBS, Gibco, Shanghai, China), 1% (v/v) non-essential amino acid, and penicillin/streptomycin (Gibco, Shanghai, China). All cells were maintained at 37 °C, 5% CO_2_ in a humidified atmosphere. All chemicals used in this study including Trichostatin A (TSA) and Decitabine (DAC) were acquired from Sigma-Aldrich (St. Louis, Missouri, USA).

### Clinical samples

Primary osteosarcoma tissues were acquired from biopsies in 20 patients before administration of neo-adjuvant chemotherapy, and their corresponding adjacent normal bone tissues were obtained after surgical resection at Tongren Hospital, School of Medicine, Shanghai Jiao Tong University between December 2017 and March 2020. All isolated tissues were rapidly frozen in liquid nitrogen and then stored at -80 °C for further experiments. The pathology of the isolated tissues was independently characterized by two pathologists. Written informed consent was obtained from each patient before enrollment, and all study protocols were approved by the institutional ethics review board of Tongren Hospital, School of Medicine, Shanghai Jiao Tong University.

### Bioinformatic analysis

The publicly accessible R2 Genomics Platform (https://hgserver1.amc.nl/cgi-bin/r2/main.cgi) was used for survival analysis and gene correlation. In the present study, the Genome-wide gene expression profiling GSE42352 was used for detailed analysis.

### Methylation-specific polymerase chain reaction

IRF7 promoter methylation was determined by methylation-specific PCR with bisulfite-converted DNA. Bisulfite modification of DNA to convert unmethylated cytosine residues to uracil was conducted with the CpGnome DNA Modification Kit following the protocol from the manufacturer (Sigma-Aldrich, Shanghai, China). The view of the promoter context of IRF7 was available at USCS Genome Browsers (http://genome.uscs.edu). MSP primers were designed by the online tool MethPrimer (http://www.urogene.org/methprimer/). The PCR products were separated on a 6% non-denaturing polyacrylamide gel.

### Quantitative real-time PCR

Total RNA was purified using TRIzol Reagent and 1 μg of total RNA was reversely transcribed to cDNA by PrimeScript RT reagent Kit (Takara, Tokyo, Japan) according to the manufacturer's instructions. qRT-PCR was performed on a ABI 7500 detection system using a SuperScript III Platinum SYBR Green One-Step qRT-PCR kit (Invitrogen, Carlsbad, CA, USA) according to the manufacturer's instructions. Ct values of target genes were normalized to that of housekeeping gene β-Actin. Specific qPCR primers (5' to 3') used in this study were shown as follows:

- *IRF1* forward: ATGCCCATCACTCGGATGC, *IRF1* reverse: CCCTGCTTTGTATCGGCCTG;
- *IRF2* forward: CATGCGGCTAGACATGGGTG, *IRF2* reverse: GCTTTCCTGTATGGATTGCCC;
- *IRF3* forward: AGAGGCTCGTGATGGTCAAG, *IRF3* reverse: AGGTCCACAGTATTCTCCAGG;
- *IRF4* forward: GCTGATCGACCAGATCGACAG, *IRF4* reverse: CGGTTGTAGTCCTGCTTGC;
- *IRF5* forward: GGGCTTCAATGGGTCAACG, *IRF5* reverse: GCCTTCGGTGTATTTCCCTG;
- *IRF6* forward: GGCTGCCGACTCTTCTATGG, *IRF6* reverse: CCTGGGAATTTGACCTGCTCC;
- *IRF7* forward: GCTGGACGTGACCATCATGTA, *IRF7* reverse: GGGCCGTATAGGAACGTGC;
- *IRF8* forward: ATGTGTGACCGGAATGGTGG, *IRF8* reverse: AGTCCTGGATACATGCTACTGTC;
- *SLC2A1* forward: ATTGGCTCCGGTATCGTCAAC, *SLC2A1* reverse: GCTCAGATAGGACATCCAGGGTA;
- *HK2* forward: TTGACCAGGAGATTGACATGGG, *HK2* reverse: CAACCGCATCAGGACCTCA;
- *PFKL* forward: GTACCTGGCGCTGGTATCTG, *PFKL* reverse: CCTCTCACACATGAAGTTCTCC;
- *ALDOA* forward: GCTGTCACTGGGATCACCTTC, *ALDOA* reverse: GCTCGGAGTGTACTTTCCTTGA;
- *PGK1* forward: GAACAAGGTTAAAGCCGAGCC, *PGK1* reverse: GTGGCAGATTGACTCCTACCA;
- *ENO1* forward: TGGTGTCTATCGAAGATCCCTT, *ENO1* reverse: CCTTGGCGATCCTCTTTGG;
- *PKM2* forward: ATAACGCCTACATGGAAAAGTGT, *PKM2* reverse: TAAGCCCATCATCCACGTAGA;
- *LDHA* forward: TTGACCTACGTGGCTTGGAAG, *LDHA* reverse: GGTAACGGAATCGGGCTGAAT;
- *ACTB* forward: CATGTACGTTGCTATCCAGGC, *ACTB* reverse: CTCCTTAATGTCACGCACGAT.

### Cell transfection

For overexpression of IRF7, a full-length human IRF7 cDNA lacking its 3'-untranslated Regions (3'-UTR) was synthesized by GenePharma (Shanghai, China) and inserted into the pcDNA3.1 (+) plasmid as reported elsewhere. OS cells in the logarithmic growth phase were seeded into 6-well plates at a density of 3-5 × 10^5^ cells per well, followed by transfection using Lipofectamine 2000 (Thermo Fisher Scientific, Waltham, MA, USA) according to the manufacturer's protocol. For knockdown of IRF7, pLKO empty vector and pLKO-IRF7-knockdown expressing lentivirus were generated and used for infecting KHOS and MG63 cells. Control and IRF7-silencing stable cell lines were then screened and used for further experiments.

### Western blotting analysis

OS cells were lysed in ice-cold lysis buffer to acquire total protein. Protein concentration of each treatment group or individual cells was determined with the BCA protein assay kit (Thermo Scientific, MA, USA). Equal amounts of protein were electrophoretically separated on a sodium dodecyl sulfate-polyacrylamide gel, followed by transfer to a nitrocellulose membrane. Membranes were then blocked with 5% (m/v) non-fat milk diluted in TBS containing 0.1% Tween-20 (TBST) and incubated with following primary antibodies: IRF7 (ab226836; 1:1000 dilution; Abcam, Cambridge, UK), PKM2 (ab85555; 1:1000 dilution; Abcam, Cambridge, UK), and β-actin (ab8226; 1:2000 dilution; Abcam, Cambridge, UK). After incubation with a second antibody, detection of blots was developed with the ECL Plus Western Blotting Detection System (GE Healthcare, USA) for antibody-conjugated with HRP.

### Glucose and lactate level

OS cells transfected with IRF7 overexpression plasmid or shRNA were seeded at a density of 5 × 10^6^ per 10 cm dish. After 24 hours, the culture medium was collected. Glucose uptake and lactate production were detected using the Glucose ColorimetricFluorometric Assay Kit (Catalog K606-100, BioVision, USA) and the Lactate Colorimetric Assay Kit (Catalog K627-100, BioVision, USA) according to the manufacturer's instructions, respectively. Total cell protein level was used for data normalization. The experiment was performed at least in triplicate.

### Measurement of extracellular acidification rate and oxygen consumption rate

The real-time glycolytic rate and mitochondrial metabolism was monitored by measuring extracellular acidification rate (ECAR) and oxygen consumption rate (OCR), respectively. The Seahorse XF96 metabolic flux analyzer (Seahorse Biosciences, Billerica, MA, USA) was used to detect ECAR and OCR as reported elsewhere. In brief, 2 × 10^4^ indicated OS cells were seeded into each well of the Seahorse XF 96 cell culture microplate. For ECAR detection, 10 mM glucose,1 mM oligomycin, and 100 mM 2-deoxyglucose (2-DG) were sequentially injected into the wells at the indicated time points. For OCR measurement, 1 mM oligomycin, 1 mM p-trifluoromethoxy carbonyl cyanide phenylhydrazone (FCCP) and 2 mM rotenone plus antimycin A (R&A) were sequentially injected into the wells at the indicated time points. Three technical replicates for each group were performed.

### Cell apoptosis assay

Cell apoptosis was examined by Apo-ONE® Homogeneous Caspase-3/7 Assay (G7790, Promega, USA). Briefly, OS cells with indicated treatment were seeded in a 96-well culture plate at a density of 1 × 10^4^ cells per well. After serum starvation for 24 h, cells were subjected to the detection of Caspase-3/7 Assay according to the manufacturer's protocol. Three technical replicates were performed.

### Cell cycle analysis

IRF7-overexpressing or knockdown OS cells were routinely trypsinized, washed with cold PBS three times, and fixed with 75% ethanol at 4 °C overnight. The next day, OS cells were stained with propidium iodide (PI, Beyotine, Shanghai, China), followed by flow cytometry analysis (BD Biosciences, CA). For statistical analysis, DNA histograms were analyzed with modified software. The assay was performed three times independently.

### Plate colony formation assay

Cells were seeded into 6-well plates at low density and were cultured with 10% FBS, 37 °C in a humidified 5% CO_2_ atmosphere. Two weeks later, colonies were fixed with methanol, stained with crystal violet, and counted under a microscope. All experiments were performed in triplicates.

### Immunohistochemistry

Firstly, xenograft tumor tissues were subjected to deparaffinization and rehydration, followed by antigen retrieval with citrate buffer (Sangon, Shanghai, China) and endogenous peroxidase blocking with hydrogen peroxide. Nonspecific binding was blocked by incubation with 10% (v/v) bovine serum albumin (BSA, Sangon, Shanghai, China). Next, the primary antibody against Ki67 (ab15580; 1:200 dilution; Abcam, Cambridge, UK) was deposited, immunoreactivity was detected with DAB chromogen and tumor sections were counterstained with hematoxylin. Negative controls were also conducted to omit the primary antibody.

### Luciferase reporter assay

The promoter region of *PKM2* gene was amplified from the genomic DNA and subcloned into the pGL4-Basic luciferase reporter plasmid (Promega, USA). Meanwhile, a mutant plasmid (*PKM2* promoter of predicted IRF7 binding sites) was also generated and subcloned into the pGL4-Basic vector (Promega, USA). The wild type (WT) and mutant constructs were confirmed by sequencing. Saos-2 cells (2.0 × 10^5^) were 24-well plates and transfected with luciferase reporter plasmids and a pRL-TK Renilla luciferase reporter plasmid at 50 ng/well using X-tremeGENE 9 (Roche, USA) according to the manufacturer's protocol. After transfection for 24 h, cells were collected and lysed with passive lysis buffer (Promega, USA), and luciferase activity was detected by the dual-luciferase reporter assay system (Promega, USA) according to the manufacturer's protocols. The experiment was performed in triplicate.

### Animal experiment

BALB/c nude mice (Male 6-week old) were obtained from the Chinese Academy of Sciences (Shanghai, China). Mice were housed in groups of 5 mice in standard polypropylene cages in 12-h light-dark cycle and were bred and maintained in a specific pathogen-free facility with free access to food and water. Tumorigenicity was analyzed by subcutaneous injection with 2 × 10^6^ OS stable cells and their corresponding normal cells. Tumor growth was monitored every 3-4 days and tumor volumes were calculated from the length (L) and the width (W) via the following formula: tumor volume (mm^3^) =LM^2^/2. Four weeks later, the mice were killed under anesthesia and the tumors were collected and fixed in 4% paraformaldehyde. All mouse experiments were conducted in accordance with standard operating procedures approved by the Ethical Committee of Tongren Hospital, School of Medicine, Shanghai Jiao Tong University.

### Statistical analysis

Results are presented as mean ± standard deviation. All statistical analyses were conducted with GraphPad Prism 5 (San Diego, CA) software or SPSS V.16.0 for Windows (IBM). Student's t-test or one-way ANOVA was used for comparisons between two groups. Overall survival was calculated by the Kaplan-Meier method and analyzed by the log-rank test. P less than 0.05 were flagged as statistically significant.

## Results

### IRF7 is downregulated in OS and correlates a better prognosis

**Figure 1:**
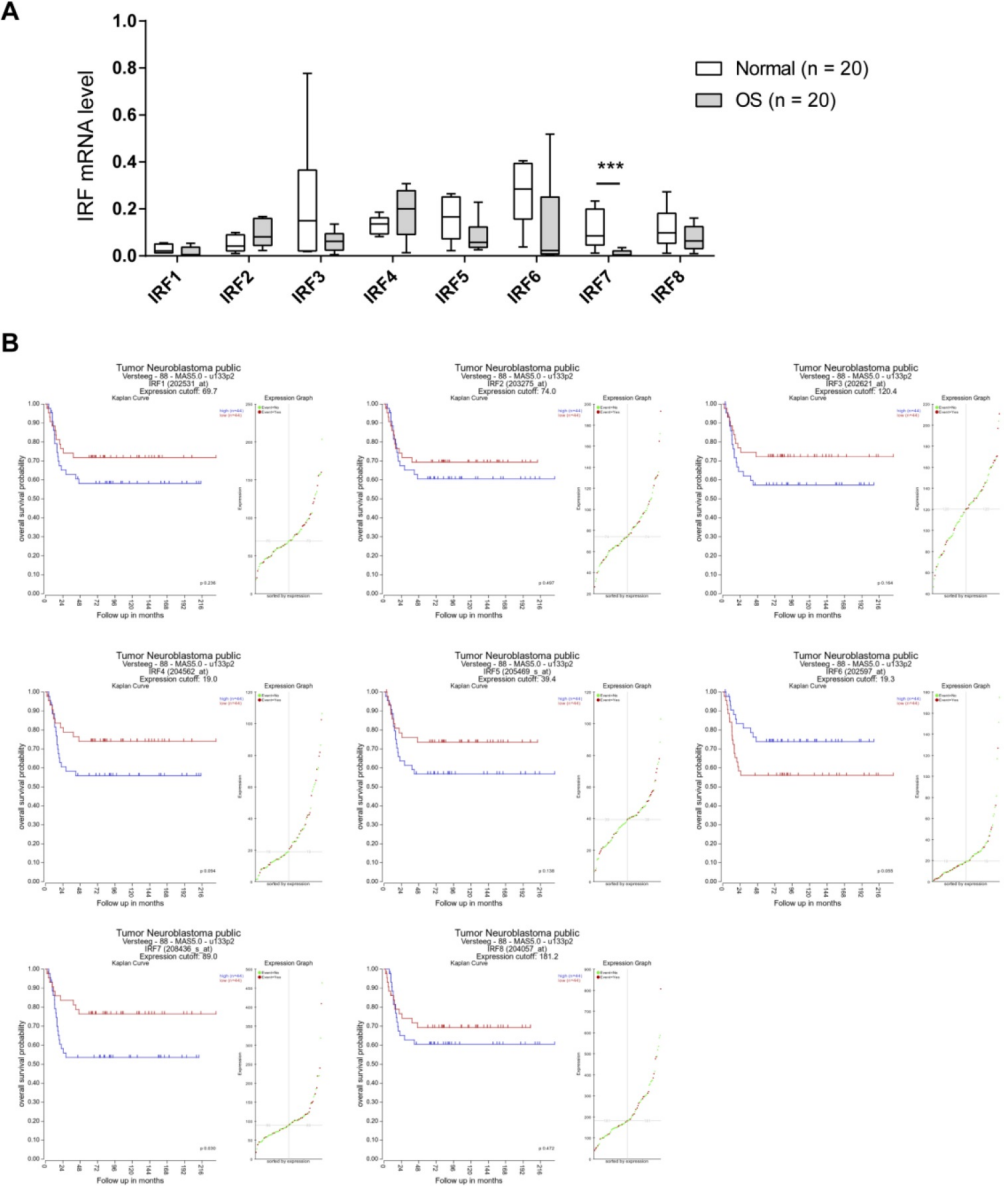
** IRF7 is downregulated in OS and correlates a better prognosis. (A)** The mRNA level of IRF family members in 20 OS and 20 corresponding normal control tissues was analyzed by real-time qPCR. **(B)** Kaplan-Meier curve showed the correlation between the expression of IRF family members and the overall survival of 88 OS patients. ***P < 0.01.

To investigate the expression pattern of IRF family members in OS, we analyzed their expression level in 20 OS and 20 corresponding normal control tissues by real-time qPCR. As shown in **Fig. 1A**, IRF3, IRF5, IRF6, and IRF7 were downregulated in OS tissues in comparison to normal control tissues. Notably, only IRF7 exhibited a significant difference. Next, we determined the prognostic value of IRF family members by searching the R2 database (https://hgserver1.amc.nl/cgi-bin/r2/main.cgi). A cohort with 88 samples was used for analysis. By Kaplan-Meier curve analysis and the median expression value as a cutoff, we revealed that all the IRF family members had an inverse correlation with OS patients' prognosis (**Fig. 1B**). Interestingly, only IRF7 reached a statistically significance. Therefore, we focused on IRF7 for detailed analysis.

### Aberrant methylation contributes to the silencing of IRF7 in OS

**Figure 2:**
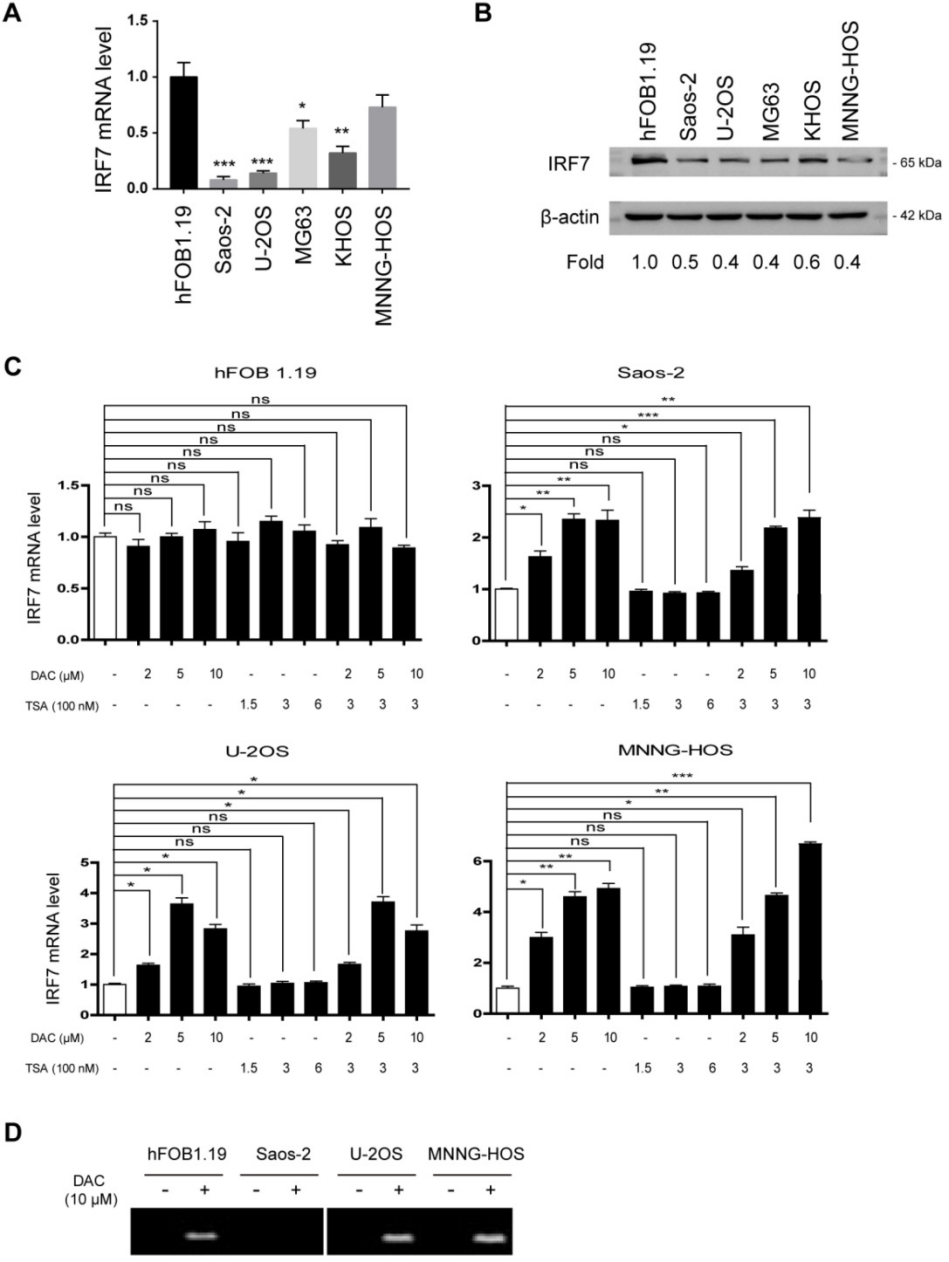
** Aberrant methylation contributes to the silencing of IRF7 in OS. (A)** The mRNA level of IRF7 in 5 OS cell lines and the nonmalignant hFOB1.19 cells was analyzed by real-time qPCR.** (B)** The protein level of IRF7 in 5 OS cell lines and the nonmalignant hFOB1.19 cells was analyzed by Western blotting. **(C)** The mRNA level of IRF7 in hFOB1.19, Saos-2, U2OS, and MNNG-HOS cells upon treatment with different concentrations of DAC and TSA was analyzed by real-time qPCR. **(D)** Representative methylation-specific polymerase chain reaction (PCR) at IRF7 promoter in the presence or absence of DAC treatment. *P < 0.05; **P < 0.01; ***P < 0.001.

By real-time qPCR analysis of IRF7 expression in OS cell lines, we found that its expression was significantly downregulated in OS cells compared with the nonmalignant hFOB1.19 cells (**Fig. 2A**). Furthermore, we confirmed this finding by Western blotting analysis (**Fig. 2B**). To uncover the reason for IRF7 downregulation in OS, we tested the possibility of an epigenetic mechanism. To achieve this, we treated OS cells and hFOB1.19 cells with different concentrations of DAC (a DNA methyltransferase inhibitor) and histone deacetylase inhibitor Trichostatin A (TSA), respectively. The result showed that the expression level of IRF7 in three OS cell lines (Saos-2, U2OS, and MNNG-HOS) was markedly increased by DAC treatment alone or in combination with TSA in a dose-dependent manner, while TSA treatment had no significant effect on IRF7 expression (**Fig. 2C**). Of note, neither DAC nor TSA affected IRF7 expression in hFOB1.19 cells. Additionally, IRF7 promoter methylation was determined by methylation-specific PCR with bisulfite-converted DNA. As a result, IRF7 promoter was were generally methylated in Saos-2, U2OS, and MNNG-HOS cells except for hFOB1.19 and the methylation status was largely reduced by addition of NAC (**Fig. 2D**). Taken together, these data hint that aberrant methylation might a reason for IRF7 downregulation in OS.

### IRF7 inhibits the Warburg effect in OS

**Figure 3:**
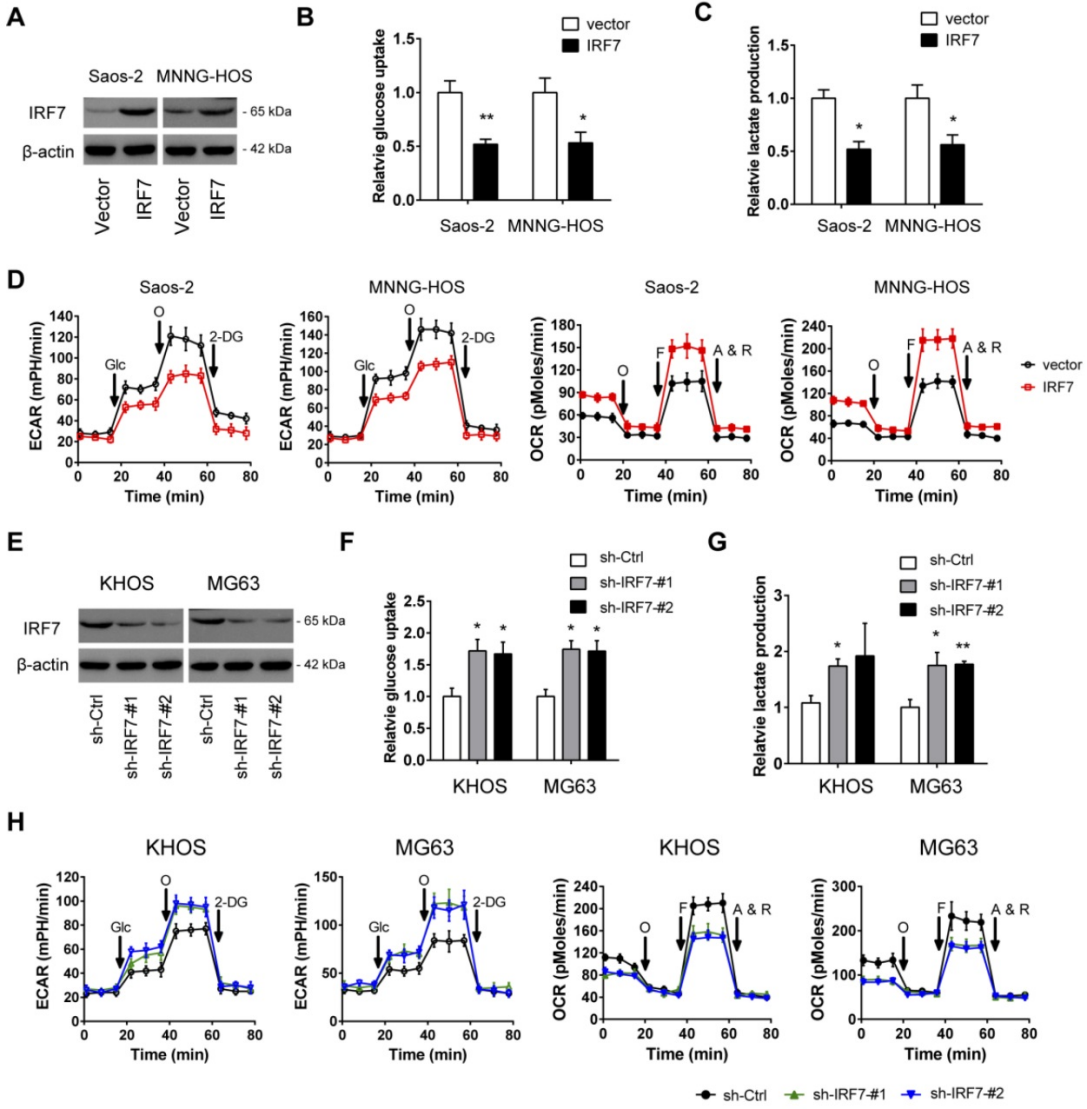
** IRF7 inhibits the Warburg effect in OS. (A)** The overexpression efficiency of IRF7 in Saos-2 and MNNG-HOS cells was detected by Western blotting.** (B-D)** Measurement of the roles of IRF7 overexpression on the glucose uptake **(B)**, lactate secretion **(C)**, extracellular acidification rate and oxygen consumption rate **(E)** in Saos-2 and MNNG-HOS cells. **(E)** The knockdown efficiency of IRF7 in KHOS and MG63 cells was detected by Western blotting.** (F-H)** Measurement of the roles of IRF7 knockdown on the glucose uptake **(F)**, lactate secretion **(G)**, extracellular acidification rate and oxygen consumption rate **(H)** in KHOS and MG63 cells. *P < 0.05; **P < 0.01.

To determine whether IRF7 affects the malignant phenotypes of OS cells, we first carried out gain-of-function studies in two cell lines (Saos-2 and MNNG-HOS) with relatively lower expression of IRF7. The overexpression efficiency of IRF7 was displayed in **Fig. 3A**. During the cell culture, we found that the acidification of the over-IRF7 Saos-2 and MNNG-HOS cells was much slower than their vector control cells, suggesting a role of IRF7 in lactate production and glucose metabolism. To test this possibility, we determined the glucose level and lactate level in the culture medium. As a result, IRF7 overexpression significantly reduced glucose utilization (**Fig. 3B**) and lactate release (**Fig. 3C**) in Saos-2 and MNNG-HOS cells. To further confirm this observation, we used the Seahorse XF96 Bioenergetic Analyzers to measure the extracellular acidification rate (ECAR) and oxygen consumption rate (OCR). As shown in **Fig. 3D**, IRF7 overexpression reduced ECAR and increased OCR, indicating a role of IRF7 in shifting aerobic glycolysis to mitochondrial oxidative phosphorylation. Moreover, we performed gain-of-function studies in KHOS and MG63 cells (**Fig. 3E**). In contrast, IRF7 knockdown boosted the glycolytic capacity of OS cells as evidenced by increased glucose uptake, increased lactate production, elevated ECAR, and decreased OCR (**Fig. 3F-H**). Taken together, IRF7 plays a suppressive role in the Warburg effect in OS cells.

### IRF7 suppresses OS cell proliferation and promotes cell apoptosis

**Figure 4:**
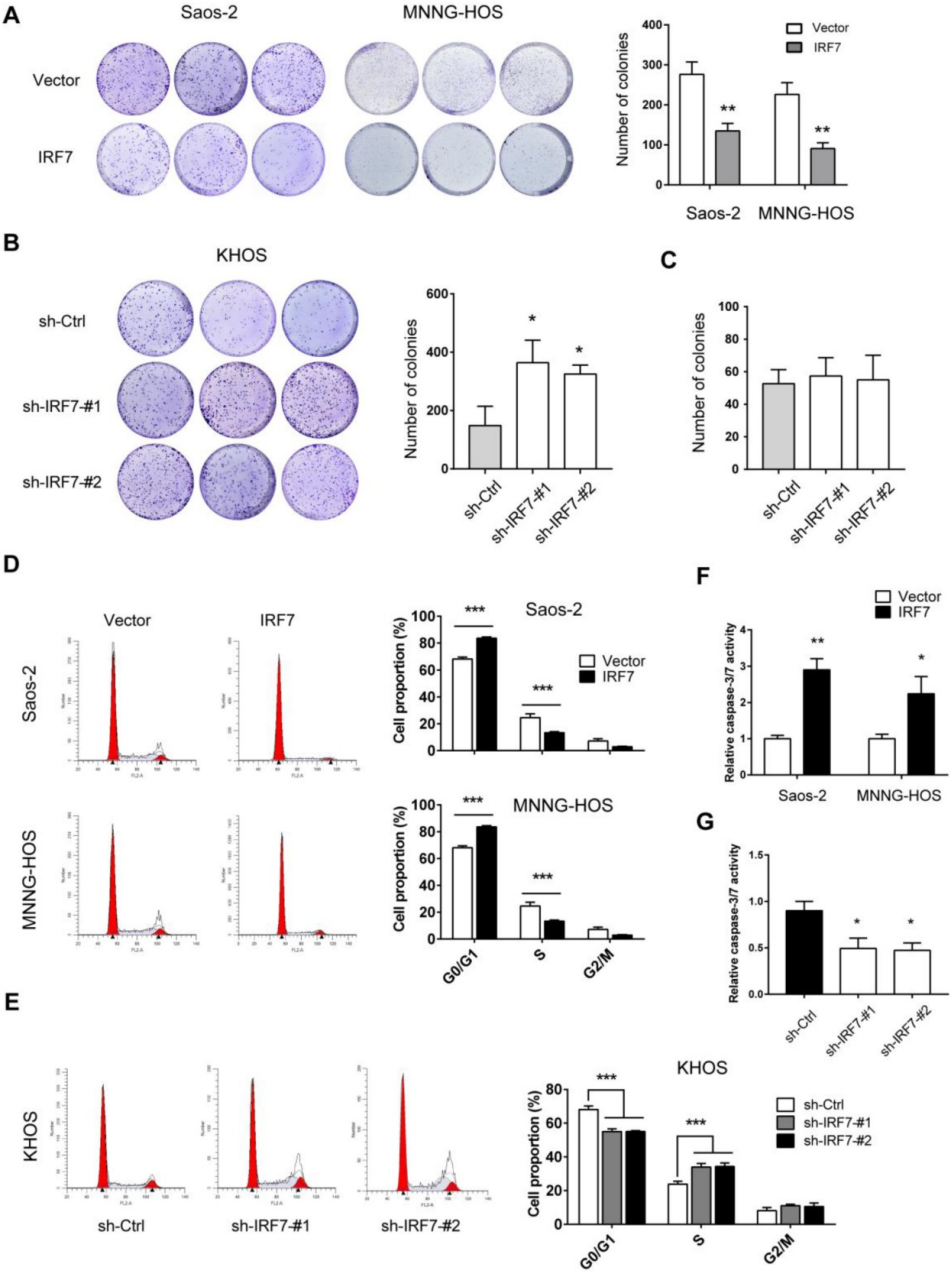
** IRF7 suppresses OS cell** proliferation and **promotes cell apoptosis. (A)** The growth rate of over-vector and over-IRF7 Saos-2 and MNNG-HOS was monitored by the plate colony formation assay. **(B)** The growth rate of sh-Ctrl and sh-IRF7 KHOS cells in the complete medium was determined by the plate colony formation assay. **(C)** In the culture medium, glucose was replaced by galactose; then, the growth rate of sh-Ctrl and sh-IRF7 KHOS cells in culture was determined by the plate colony formation assay. **(D)** The cell cycle status of over-vector and over-IRF7 Saos-2 and MNNG-HOS cells was analyzed by flow cytometry.** (E)** The cell cycle status of sh-Ctrl and sh-IRF7 KHOS cells was analyzed by flow cytometry. **(F)** The cell apoptosis status of over-vector and over-IRF7 Saos-2 and MNNG-HOS upon serum starvation for 24 h was determined by the Caspase3-7 activity assay. **(G)** The cell apoptosis status of sh-Ctrl and sh-IRF7 KHOS upon serum starvation for 24 h was determined by the Caspase3-7 activity assay. *P < 0.05; **P < 0.01; ***P < 0.001.

To further investigate the effect of IRF7 on OS cell proliferation, we performed plate colony formation experiment. As shown in **Fig. 4A**, IRF7 overexpression significantly inhibited Saos-2 and MNNG-HOS cell growth as demonstrated by less number of colonies. In contrast, IRF7 knockdown promoted KHOS cell proliferation (**Fig. 4B**). Notably, if we grew KHOS in galactose instead of glucose, the promotive effect of IRF7 knockdown in cell proliferation was compromised, suggesting a glycolysis-dependent role of IRF7 in OS cells (**Fig. 4C**). Moreover, we investigated whether IRF7 affects cell cycle progression is OS cells. As a result, cell cycle was progressed by IRF7 knockdown in KHOS cells while IRF7 overexpression induced cell cycle arrest in Saos-2 and MNNG-HOS cells (**Fig. 4D** and** 4E**). In addition, we tested the cell apoptosis status upon genetic manipulation of IRF7. We grew OS cells without serum for 24 h and cells were harvested for measurement of Caspase-3/7 activity. Expectedly, IRF7 overexpression promoted Saos-2 and MNNG-HOS cell apoptosis (**Fig. 4F**), while IRF7 knockdown reduced KHOS cell apoptosis (**Fig. 4G**).

### IRF7 attenuates OS tumor growth *in vivo*

**Figure 5:**
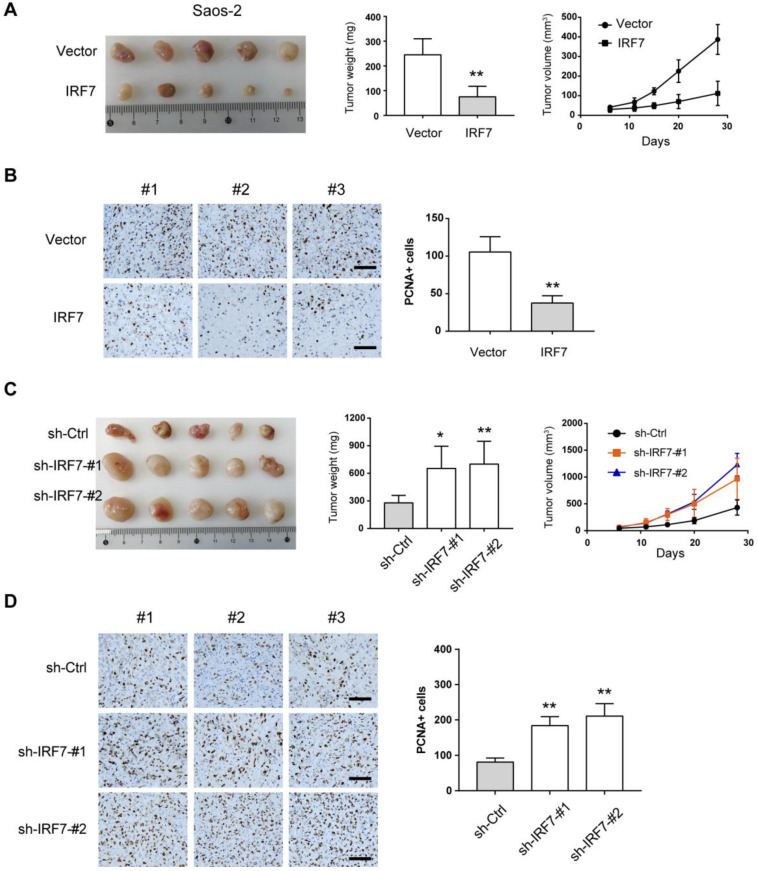
** IRF7 attenuates OS tumor growth *in vivo.* (A)** Measurement of the role of IRF7 overexpression on the subcutaneous xenograft tumors from Saos-2 cells; tumor weight and tumor volume were shown. **(B)** Ki67 staining was performed in the subcutaneous xenograft tumors from Saos-2 cells. **(C)** Measurement of the role of IRF7 knockdown on the subcutaneous xenograft tumors from KHOS cells; tumor weight and tumor volume were shown. **(D)** Ki67 staining was performed in the subcutaneous xenograft tumors from KHOS cells. *P < 0.05; **P < 0.01.

Next, we used the subcutaneous xenograft model to investigate the effects of IRF7 knockdown or overexpression on OS cell growth. As shown in **Fig. 5A**, IRF7 overexpression significantly inhibited tumor growth of Saos-2 cells as demonstrated by reduced tumor weight and tumor volume. By immunohistochemical analysis, we found that xenograft tumors from over-IRF7 Saos-2 cells had less positive staining of the proliferation index Ki67 compared with that from over-vector Saos-2 cells (**Fig. 5B**). In contrast, IRF7 knockdown rapidly enhanced the growth of xenograft tumors from KHOS cells as supported by higher tumor weight, larger tumor volume, and more positive staining of Ki67 (**Fig. 5C-D**). Collectively, these data above suggest that IRF7 acts as a tumor suppressor in OS.

### IRF7 transcriptionally suppresses PKM2 expression in OS

**Figure 6:**
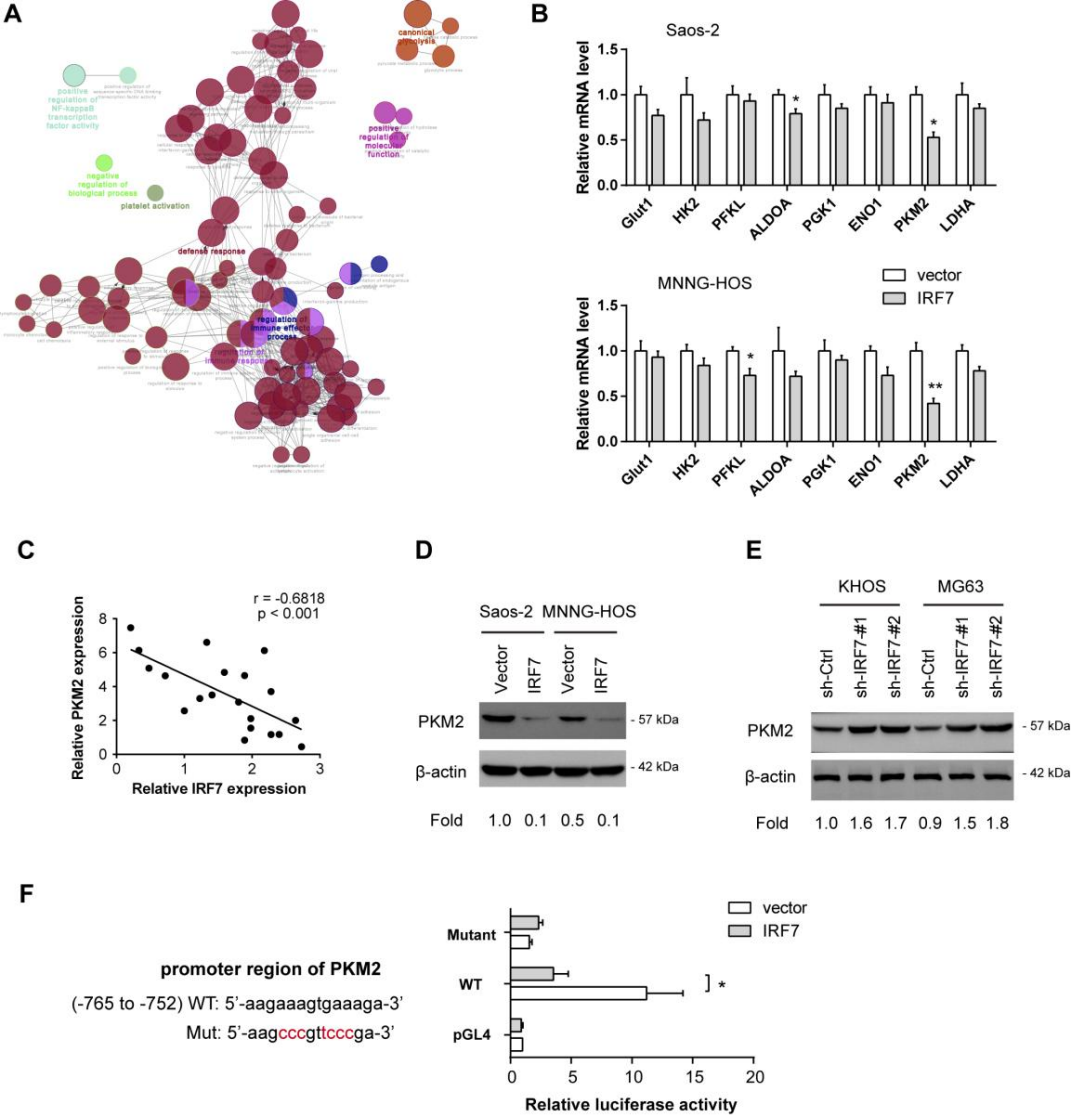
** IRF7 transcriptionally suppresses PKM2 expression in OS. (A)** Annotation of IRF7-associated genes in OS; data were acquired from R2 and the figure was generated by Cytoscape software. **(B)** Measurement of the roles of IRF7 overexpression on the expression of glycolytic genes in Saos-2 and MNNG-HOS cells.** (C)** Correlation analysis of gene expression in 20 OS patient samples between IRF7 and PKM2. **(D)** The effect of IRF7 overexpression on PKM2 expression in Saos-2 and MNNG-HOS cells was detected by Western blotting.** (E)** The effect of IRF7 knockdown on PKM2 expression in KHOS and MG63 cells was detected by Western blotting.** (F)** Luciferase activity of PKM2 gene promoter reporters in Saos-2 cells transfected with IRF7 or empty vector. Red sites indicate the putative IRF7-binding sites; WT represents wild type. *P < 0.05; **P < 0.01.

Finally, we aimed to decipher the molecular mechanism by which IRF7 regulates the Warburg effect. By annotation of IRF7-associated genes in OS, we found that IRF7 was profoundly involved in diverse biological processes, such as regulation of immune effector process, regulation of immune response, defense response, positive regulation of NF-kappaB transcription factor activity, and canonical glycolysis (**Fig. 6A**). By real-time qPCR analysis, we analyzed the effect of IRF7 overexpression on the expression of glycolytic genes in Saos-2 and MNNG-HOS cells. As a result, PKM2 was significantly reduced by IRF7 overexpression in both Saos-2 and MNNG-HOS cells, suggesting that PKM2 might be the molecular transcriptional target of IRF7 in OS cells (**Fig. 6B**). In clinical samples, IRF7 expression was closely associated with PKM2 in 20 OS tissues (**Fig. 6C**). Western blotting analysis showed that IRF7 overexpression downregulated PKM2 expression in Saos-2 and MNNG-HOS cells (**Fig. 6D**), while IRF7 knockdown increased PKM2 protein level in KHOS and MG63 cells (**Fig. 6E**). To determine whether PKM2 can be transcriptionally suppressed by IRF7, we predicted the potential binding site of IRF7 in the PKM2 gene promoter with JASPAR. By luciferase reporter assay, we revealed that IRF7 can significantly inhibit PKM2 transcription in Saos-2 cells (**Fig. 6F**). Therefore, these findings above indicate that IRF7 might suppress the Warburg effect via transcriptional regulation of PKM expression in OS.

## Discussion

Identification of the relationship between reprogrammed energy metabolism and OS is of paramount importance to uncover OS pathogenesis and provide new insights in the targeted therapies of OS. Previously, several reports have evaluated a few oncogenes or tumor suppressors in the glycolytic metabolism of OS cells, such as SLIT2/ROBO1 7, CircECE1 23, S1P 24, and LncRNA KCNQ1OT1 25. These genes were found to be associated with OS cell proliferation, migration, invasion, cellular transformation, and cell apoptosis. Due to the poor clinical outcome of OS and the limitations of current treatment, the present study determined the role of IRF family members during tumor metabolic reprogramming.

For the first time, we identified that IRF7 but not other members of the IRF family were significantly decreased in OS tissues and OS cell lines. Moreover, Higher IRF7 expression was associated with a better prognosis. These findings indicate that IRF7 may act as a tumor suppressor during the development of OS. In this study, we provided evidence that methylation of the IRF7 promoter region is responsible for its downregulation in OS. Indeed, the promoter region of IRF7 gene contains a putative CpG island flanking the TATA box. In line with our findings, methylation of the IRF7 promoter region has also been detected in immortalized fibroblasts from Li-Fraumeni syndrome and in lung cancer cells 26, 27. Previously, IRF7 has been demonstrated to play important roles in the tumor initiation and progression of several types of cancers. For instance, in a mouse model of spontaneous bone metastasis, genetic silencing of IRF7 significantly promotes breast cancer bone metastasis via immune escape 28; in lung cancer cells, knockdown of IRF7 increases sensitivity to oncolytic viruses 29; moreover, IRF7 deficiency leads to significant accumulation of granulocytic myeloid-derived suppressor cells (G-MDSCs), and therefore promoted tumor growth and metastasis in mice 30. Consistent with these observations above, data from our study showed that IRF7 is profoundly implicated in the glycolytic metabolism and tumor growth of OS cells. Using a nude mouse xenograft tumor model, silencing of IRF7 can significantly attenuate tumor growth *in vivo*. Of note, the tumor-suppressive role of IRF7 in tumor growth was largely dependent on the Warburg effect as hijacking glycolysis with galactose largely abrogated cell proliferation induced by IRF7 knockdown. Apart from tumor-suppressive roles, IRF7 also plays several oncogenic properties. For instance, IRF7 can be induced and activated by Epstein-Barr virus (EBV) and IRF7 reversely induces expression of the principal oncoprotein LMP1 of EBV to form positive feedback to potentiate oncogenic effects in a variety of malignancies 31. Therefore, the roles of IRF7 in human cancers might be tumor-specific.

Accumulated studies have shown that IRF7 acts as an important transcription factor to participate in many aspects of the immune responses, such as immune cell development, differentiation, and responses to pathogens 32. For example, in multiple models of allergic asthma, IRF7 induces the expression of Bcl11b and increases the expansion and function of Type 2 innate lymphoid cells (ILC2s) 33. In this study, we revealed that PKM2 is a downstream transcriptional target of IRF7 in regulating the glycolytic metabolism of OS cells. In OS cells, genetic silencing of PKM2 can efficiently inhibit cell proliferation and induce G1 cell cycle arrest and cell apoptosis, which was accompanied by decreased expression levels of cyclin D1 and Bcl-2 as well as increased expression levels of Bax, cleaved-caspase-3, and cleaved-PARP 34. Importantly, the tumor-promoting effects of PKM2 were largely glysolyis-dependent 35. Therefore, our findings provide the reasonability for targeting the IRF7-PKM2 axis in OS. Nevertheless, several limitations in this research should be mentioned. Firstly, there could be other molecular targets of IRF7 that are involved in the Warburg effect of OS cells. Indeed, we cannot fully rule out the possibility that IRF7-dependent glycolysis might be linked to inflammatory pathways, such as NF-kappaB signaling. However, our results, at least to some extent, confirmed that IRF7 transcriptionally suppressed PKM2 expression to inhibit tumor glycolysis. Secondly, most experiments were performed in cells and nude mice, the situation in human samples is encouraged to introduce.

## Conclusion

Taken together, our study is the first to reveal that IRF7 was downregulated in OS and suppressed the tumor progression and the Warburg effect in OS cells by transcriptional suppression of PKM2. The research has significant implications for our understanding of OS pathogenesis and indicates that IRF7 might be a hopeful therapeutic target for OS treatment via interfering with cancer metabolic processes.
