# IRF7 inhibits the Warburg effect via transcriptional suppression of PKM2 in osteosarcoma: Erratum

**DOI:** 10.7150/ijbs.79598

**Published:** 2022-11-10

**Authors:** Zhikun Li, Mei Geng, Xiaojian Ye, Yunhan Ji, Yifan Li, Xiangyang Zhang, Wei Xu

**Affiliations:** 1 Department of Orthopedics, Tongren Hospital, School of Medicine, Shanghai Jiao Tong University, Shanghai 200336, China.; 2 Department of Oncology, Rui Jin Hospital Affiliated to Shanghai Jiao Tong University School of Medicine, Shanghai 200025, China.

After the publication of our article, we noted one error in Fig. 1B. The Kaplan-Meier survival curve is about neuroblastoma instead of osteosarcoma. After careful examination, we confirmed that this error was caused by carelessness when we used the R2 database (https://hgserver1.amc.nl/cgi-bin/r2/main.cgi). The corrected Fig.1B is shown as follows.

Additionally, the description of Fig. 1B was changed, as shown follows: “A cohort (GSE42352) of 88 patients with survival data was used for analysis. By Kaplan-Meier curve analysis, we revealed that several IRF family members had an association with OS patients' prognosis (**Fig. 1B**). Interestingly, higher IRF7 expression was inclined to predict a better prognosis in OS patients.”

The authors confirm that the mistake does not affect the conclusions of the study and apologize for any inconvenience caused by this mistake.

## Figures and Tables

**Figure 1:**
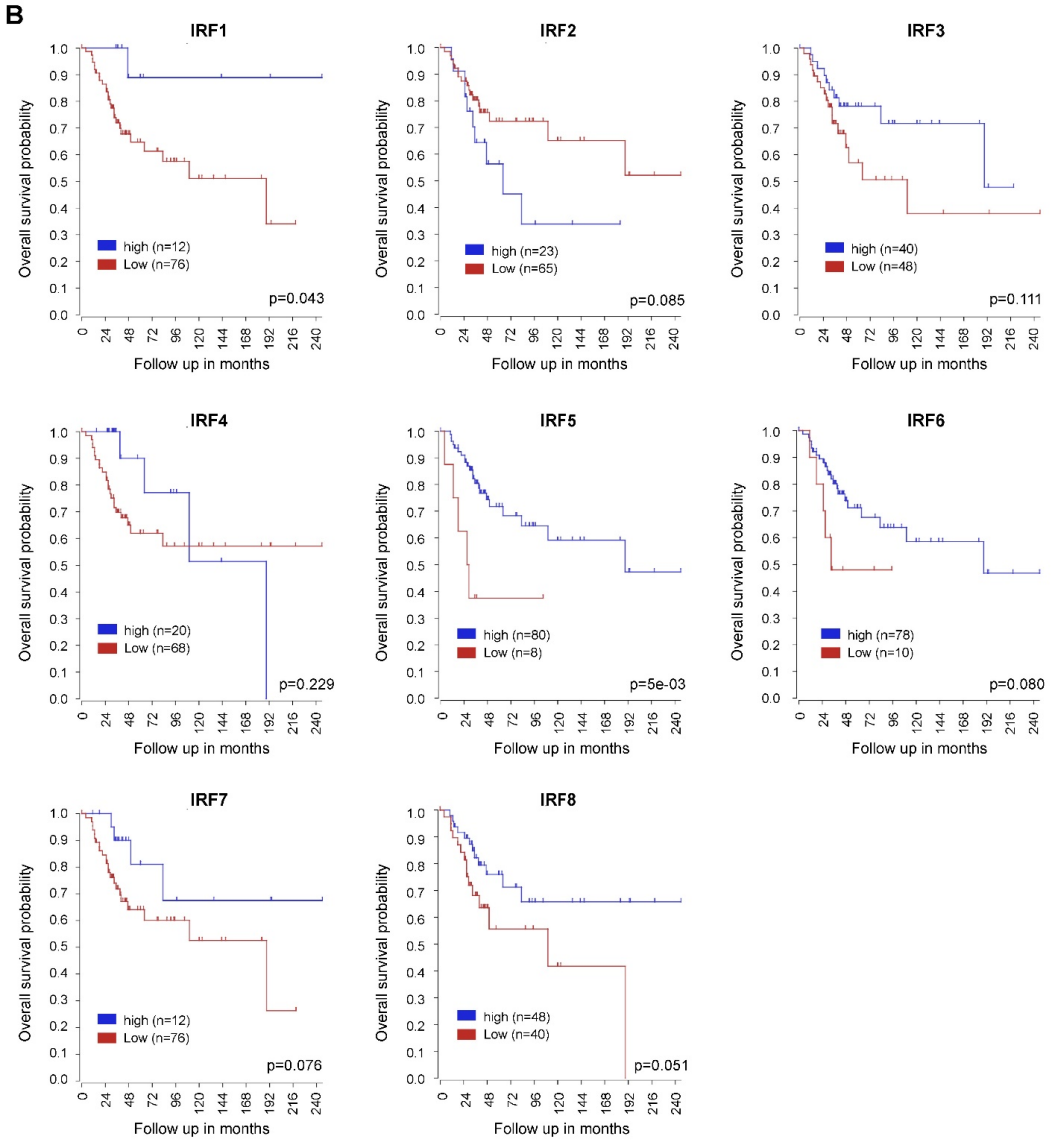
** B.** Corrected figure.

